# Sleep Disturbances in At-Risk Mental States and First Episode of Psychosis: A Narrative Review on Interventions

**DOI:** 10.3390/clockssleep5020020

**Published:** 2023-04-29

**Authors:** Lorena Marin, Armand Guàrdia, Alexandre González-Rodríguez, José Haba-Rubio, Mentxu Natividad, Elena Bosch, Noelia Domínguez, José Antonio Monreal

**Affiliations:** 1 Department of Mental Health, Mútua Terrassa University Hospital, Fundació Docència I Recerca Mutua Terrassa, 08221 Terrassa, Spain; 2 Centre for Investigation and Research in Sleep (CIRS), Centre Hospitalier Universitaire Vaudois, 1011 Lausanne, Switzerland; 3 Institut de Neurociències, UAB, CIBERSAM, 08221 Terrassa, Spain

**Keywords:** first episode of psychosis, ARMS, FEP, sleep, insomnia

## Abstract

Sleep disturbances are a common yet often overlooked symptom of psychosis that can drastically affect the quality of life and well-being of those living with the condition. Sleep disorders are common in people diagnosed with schizophrenia and have significant negative effects on the clinical course of the illness and the functional outcomes and quality of life of patients. There is a limited number of studies addressing this question in first-episode psychosis (FEP). In this narrative review, we aimed to provide an overview of sleep disorders in populations with FEP and at-risk mental states (ARMS). The review was focused on the various treatments currently used for sleep disorders, including both non-pharmacological and pharmacological treatments. A total of 48 studies were included. We found that sleep disturbances are associated with attenuated psychotic symptoms and other psychopathological symptoms in ARMSs. The association of sleep disturbances with the transition to psychosis has been poorly investigated. Sleep disturbances have an impact on the quality of life and the psychopathological symptoms of people suffering from FEP. The non-pharmacological treatments include cognitive behavioral therapy for insomnia, bright light therapy, cognitive restructuring techniques, sleep restriction therapy, basic sleep hygiene education, and the provision of portable sleep trackers. Other treatments include antipsychotics in acute phases and melatonin. The early intervention in sleep disturbances may improve overall prognosis in emerging psychosis populations.

## 1. Introduction

Sleep is a fundamental component of a person’s health. It is a well-established fact that disturbances in sleep patterns are a risk factor for various physical and psychological illnesses [1]. Some common sleep disorders include insomnia, sleep-related breathing disorders, the central disorders of hypersomnolence, circadian rhythm sleep–wake disorders, parasomnias and sleep-related movement disorders.

The prevalence of psychotic disorders varies depending on the type of disorder and the population being studied. However, several studies have suggested that psychotic disorders affect about 3% of the general population worldwide. For instance, schizophrenia, one of the most well-known psychotic disorders, is estimated to affect about 1% of the global population. This condition is associated with a total economic burden of about EUR 17.5 billion in Spain, with EUR 11 billion derived from indirect costs [2]. The main symptoms of schizophrenia are divided into positive psychotic symptoms (e.g., hallucinations, delusions and disorganized thinking), negative symptoms (e.g., social withdrawal, emotional withdrawal, blunted affect and anhedonia) and cognitive symptoms (e.g., impairment in executive functions), all of which may indicate schizophrenia when present for a duration of six months or more [3]. Other authors have also included depressive symptoms as a separate symptomatic domain. Sleep disturbances are also a frequent symptom and have been correlated with poor clinical outcomes [4].

First-episode psychosis (FEP) is more frequent among the population aged between 15 and 40 years [5]. This is why many programs have been implemented with the objective of achieving the early detection and early treatment of patients suffering from FEP. This also applies for the population with at-risk mental states (ARMS) [6].

Within the ARMS group, there are many spectrum syndromes, including attenuated psychotic symptoms (APS) and brief limited intermittent psychotic symptoms (BLIPS). There is also genetic risk, which involves a significant reduction in functioning [7]. From those patients fulfilling the ARMS criteria, approximately 22–30% will experience psychosis within 12 months of the clinical onset of their ARMS [7]. In brief, APSs are defined as attenuated or subthreshold psychotic symptoms [8]. BLIPSs are cases in which psychotic experiences are spontaneously resolved within 1 week [8]. Table 1 summarizes the main characteristics of individuals with ARMSs.

In FEP patients, sleep disturbances can have a significant impact on the course and outcome leading to an increased risk of relapse, a decreased quality of life, and impaired cognitive and social functioning [9]. In the last 10 years, several studies have suggested that sleep disturbances may also be implicated in the transition to psychosis in ARMS populations and the exacerbation of psychotic symptoms in FEP patients [10,11,12,13]; however, the nature of this relationship remains unclear.

The effective management of sleep disturbances in FEP is crucial for improving outcomes and reducing the risk of relapse.

The main aim of the present work is to review the sleep disturbances seen in patients with FEP and in individuals suffering from ARMSs. As a second objective, we aim to summarize pharmacological and non-pharmacological approaches for treating sleep disturbances in these populations.

## 2. Method

A non-systematic narrative review was carried out with the main objective of summarizing the evidence from the literature on sleep disorders related to FEP and ARMSs.

For this purpose, electronic searches were performed using PubMed, Google Scholar and ClinicalTrials.gov. Searches were conducted for papers published in English and Spanish that addressed our main objectives.

The following search terms were used: “first episode psychosis” OR “ultra high risk” OR “at risk mental state” OR “antipsychotic treatment” AND “sleep” OR “insomnia” OR “circadian” OR “sleep disturbances”.

Additional studies were included if they were published in the last 10 years and were referring to the topic of interest. Classic papers and other relevant papers were also included if they were cited in studies included in the present review.

Results obtained were divided into the following four sections:

(A) Sleep disturbances in ARMSs;
(B) Sleep disturbances in FEP;
(C) Non-pharmacological treatment;
(D) Pharmacological treatment of sleep disturbances in FEP and ARMSs.

## 3. Sleep Disturbances in ARMSs

### 3.1. Studies Including Subjective Assessment of Sleep Disturbances in ARMSs

Sleep disturbances are a common feature of psychotic disorders. They can also be present in ARMSs. The evidence suggests sleep disturbances’ potential role in the progression of symptoms; however, the association between sleep and ARMS symptoms has not been adequately investigated [14].

Shetty and collaborators [15] aimed to investigate the biological processes in at-risk individuals to elucidate the potential mechanisms underlying the development of psychosis. The authors explored the associations between sleep disturbances, chronotype and depressive and psychotic symptoms in 81 individuals with ARMSs. Both sleep disorders and chronotypes have been found to influence the psychopathology in this population. A greater preference for eveningness has been associated with increased negative symptoms in the overall population.

In 2021, Nuzum and colleagues [16] carried out a retrospective study including 795 ARMS individuals with the main aim of investigating sleep disorders in people suffering from psychotic disorders. For the assessment of psychopathology and sleep disturbances, the Clinical Records Interactive Search (CRIS) tool was used. Sleep problems, subsequent diagnoses, attenuated positive symptoms and Health of The Nation Outcome Scale scores were identified. Sixty per cent of ARMS individuals presented sleep disturbances. Although sleep problems were not associated with a higher risk of transition to psychosis, they were correlated with a shorter transition time in individuals who developed psychosis and higher follow-up scores on the Health of Nation Outcome Scale (HoNOS). The authors highlighted the need to improve sleep disturbances in these populations to reduce the severity of positive symptoms and improve functionality.

Several studies suggest that sleep disturbances are correlated with a higher severity of both positive and negative symptoms and poorer overall functioning in ARMS patients over time [14,17,18,19,20].

### 3.2. Studies Including Objective Assessment of Sleep Disturbances in ARMSs

Some studies have reported an association between sleep quality and cognition in adolescents with ARMSs. Lunsford-Avery and collaborators found that better sleep quality in ARMS individuals was associated with a steeper learning curve, thus establishing a link between sleep disturbances and cognitive deficits [21].

Similarly, other studies [22] link sleep disturbances to psychological well-being and functioning, with the treatment of these disturbances having a significant impact on the well-being of these young people. However, it has not yet been established which kind of sleep characteristics are most implicated in ARMS symptomatology [23].

In summary, sleep disturbances have been found to be associated with the presence of attenuated psychotic symptoms and other psychopathological symptoms in at-risk mental states (ARMS). Their association with the transition to psychosis should be further investigated.

## 4. Sleep Disturbances in FEP

### 4.1. Studies Including Subjective Assessment of Sleep Disturbances in FEP

In 2010, Fekih-Romdhane and colleagues [24] conducted a comparative sleep study between patients with first-episode schizophrenia (54 patients), their unaffected siblings (56 individuals) and healthy controls (61 individuals) matched with patients and siblings. Patients from these three study groups were assessed using the PSQI (Pittsburgh Sleep Quality Index) and Horne and Ostberg’s Morningness–Eveningness questionnaire. Psychopathological symptoms and functionality were assessed by means of the Positive and Negative Syndrome Scale (PANSS) and GAF respectively. The authors found that patients with emerging schizophrenia and siblings of patients suffering from psychosis presented lower sleep quality compared to healthy controls. Furthermore, patients with schizophrenia had more daytime sleepiness than healthy controls. Briefly, this study found that sleep patterns may distinguish non-psychotic siblings from healthy controls and may serve as an endophenotype for schizophrenia.

In 2017, Mythily Subramaniama’s team [25] explored the prevalence of insomnia in patients with FEP and investigated the relationship between insomnia and sociodemographic and clinical variables, as well as quality of life (QOL) and functioning. They recruited 280 FEP participants from the early psychosis care program. Data on sleep patterns, quality of life, socio-demographic data, substance use and smoking within 3 months of starting the program were registered. Insomnia was found in 22.6% of the whole sample. Older age and higher dosage of antipsychotic medication were significantly associated with a lower risk of insomnia, while alcohol consumption, current smoking and longer duration of untreated psychosis were significantly associated with a higher risk of insomnia. Insomnia was associated with significant decreases in all Quality-of-Life domains assessed in the study.

Wejie Ong et al. in 2020 [26] investigated the association between quality of life and sleep quality among patients with FEP. They recruited 280 patients from the Early Intervention of Psychosis program. The following assessment instruments were used: the Pittsburgh Sleep Quality Index (PSQI) to examine sleep quality, the WHO-QOL-BREF Quality of Life scale, the PANSS for psychotic symptoms, and the GAF to evaluate functionality. The results showed that poor sleep quality was associated with lower quality of life scores. This study suggests that the improvement of sleep quality would influence clinical improvement in patients’ quality of life.

To summarize, sleep disturbances have been found to impact quality of life and psychopathological symptoms in people suffering from FEP. Early intervention on sleep disturbances may improve overall prognosis in these psychosis population.

### 4.2. Studies Including Objective Assessment of Sleep Disturbances in FEP

In 2002, Julie Poulin and collaborators [27] investigated the sleep architecture in patients suffering from a first episode of schizophrenia who had not been treated with antipsychotics in order to evaluate the relationship between sleep parameters and clinical symptoms. Eleven acute patients who were not treated with antipsychotics were included and compared with eleven healthy controls. The patients were assessed using the Brief Psychiatric Rating Scale (BPRS), and sleep assessment was performed in individual rooms. Patients with the first episode of schizophrenia had more difficulties initiating sleep, as well as reduced REM sleep latency, but normal sleep spindle and REM densities. Positive symptoms were negatively correlated with REM sleep latency. The BPRS total score was negatively correlated with REM sleep duration and REM density. These results suggest that patients with untreated first episodes of schizophrenia may have difficulty initiating sleep but not maintaining it. The observed relationship between the REM sleep measurements and the BPRS results suggests that REM sleep physiology may share common substrates with the symptoms of psychosis.

In a 2016 systematic review, Davies et al. [4] aimed to synthesize and evaluate the available data exploring sleep in early psychosis. A total of 21 articles were included in this review. The authors found that self-reported sleep disturbances are common in early psychosis, and that they may be associated with symptoms, as well as the ability to seek help and the risk of suicide. Alterations in sleep architecture were also frequently observed and associated with symptom severity and neurocognitive deficits.

Reeve et al. assessed 60 patients with early non-affective psychosis for clinical sleep disorders (e.g., insomnia, nightmare disorder and sleep apnea) that clearly cause sleep disturbances and, thus, present targets for intervention [28]. Sleep disorders were assessed using the Diagnostic Interview for Sleep Patterns and Disorders (DISP) and supplemented with subjective sleep diaries and objective wrist actigraphy for 7 days. Based on this multimethod assessment, 80% of patients reported at least one sleep disorder, with the most common diagnoses being insomnia (50%) and nightmare disorder (48%) [28]. Most of the disorders were rated as severe in terms of chronicity, frequency and distress or impairment.

Several studies report that patients with sleep disorders had more severe paranoia, hallucinations, cognitive disorganization, depression and anxiety. Quality of life was low in patients with comorbid psychosis and sleep disorders, which suggests that the clinical management of patients with psychosis should be focused on treating sleep disturbances [29]. In more than two thirds of cases, no treatment for the sleep disorder was reported. Even when treatment had been received, it was rarely the recommended treatment for the sleep disorder [29].

Several studies have shown alterations in both macro- and microstructural parameters in the sleep of patients with schizophrenia. In 2019, Kaskie and colleagues [30,31] conducted a study assessing high-density (hd) EEG recordings in 27 patients with FEP and 23 healthy controls (HC). Several spindle parameters—amplitude, duration and density—were calculated and compared between the groups. The patients with FEP showed a reduction in spindle duration and spindle density, but not in spindle amplitude, relative to the HCs. These spindle reductions were in frontal areas and predicted the severity of negative symptoms in FEP patients. More recently, Naksidil Torun Yazıhan [32] conducted a study in 21 patients with FEP and HCs who underwent polysomnographic recordings for 2 nights, of which none were receiving pharmacological treatment. A neuropsychological assessment was performed and the PANSS was used to assess symptom severity. In this study, they found that sleep efficiency was lower in patients than in the control group, and a reduced spindle density was also found. The percentage of N3 stage sleep was found to decrease as negative symptoms increased, and the percentage of N1 stage sleep increased as the severity of negative symptoms increased.

## 5. Non-Pharmacological Treatments

A high amount of research supports the notion that sleep disorders are one of the most promising modifiable risk factors for psychosis [18,19], which has been widely reported in individuals at high clinical risk. Evidence has also shown that sleep disturbances are present from the earliest stages of the disease, even during the pre-diagnosis phase [32]. Non-pharmacological treatments are crucial in the treatment of sleep disorders in FEP patients, as well as in the management of at-risk mental state individuals.

Early interventions that target modifiable factors in ARMS individuals, such as sleep, offer the opportunity to have a greater impact on outcomes by using personalized and less harmful treatment methods [12,13,14,15,16,17,18,19,20,21,22,23,24,25,26,27,28,29,30,31,32,33,34]. On the other hand, sleep therapy is a promising intervention target for symptom improvement, given that sleep is a driving force in symptom exacerbation. Promoting healthy sleep may be a useful target for the symptom maintenance of ARMSs and PEP.

The vast majority of evidence has been accumulated in support of cognitive behavioral therapy (CBT) for insomnia, which has been demonstrated to reduce insomnia but also to improve clinical psychotic attenuation [14,35,36,37,38]. In fact, cognitive behavioral therapy for insomnia (CBTi) is the first-line treatment for adults with insomnia [39] and has been effectively adapted for people with psychosis [35,40]. CBTi has also been shown to have benefits such as improving depression and anxiety and attenuating psychotic symptoms [36,41].

Freeman and colleagues [36] assessed the efficacy of CBT for sleep problems in patients suffering from psychotic experiences. The authors conducted a prospective, blinded, randomized trial in two mental health centers in the United Kingdom. This study found that, compared to standard care, CBT was associated with reductions in insomnia in the large effect size range at week 12, thus demonstrating that CBT for insomnia could be very effective in improving sleep in patients with persistent delusions or hallucinations.

Waite and coworkers described findings on sleep problems in schizophrenia and recommendations for interventions [41]. The authors identified 12 factors potentially contributing to sleep disturbances and highlight that early interventions are capable of reducing psychotic experiences. Once again, treating insomnia was found to reduce psychotic symptoms.

Robertson et al. found that CBT for insomnia alleviates insomnia-related symptoms by using strategies such as cognitive restructuring, stimulus control and insomnia via strategies such as cognitive restructuring, stimulus control and relaxation training [42]. In their review, acupuncture is also considered a possible therapeutic option for insomnia, either in monotherapy or as an adjunct therapy to pharmacotherapy or CBT for insomnia and may lead to improvements in clinical outcomes. It also specifies the positive results observed with a constant moderate-intensity exercise regimen, which may help to improve sleep disturbances in patients with schizophrenia [42].

In 2021, Henry et al. examined the effect of fully automated digital CBT for insomnia (Sleepio) and the mediating role of sleep improvement on depressive symptoms [43]. The authors found that digital CBT significantly improved insomnia and depressive symptoms at postintervention (weeks 8–10).

The brief psychological intervention ‘Sleep Well’, which targets key sleep parameters (i.e., hyperexcitability, sleep, blood pressure and circadian rhythm), has produced promising preliminary results [38]. Waite and collaborators designed a protocol (SleepWell) as a psychological intervention designed for young people that targets the key mechanisms that regulate sleep: circadian rhythm, sleep pressure and hyperarousal [44]. It also includes worry reduction strategies, cognitive restructuring techniques and night-time relaxation methods [44].

Basic sleep hygiene education, such as avoiding caffeine or alcohol consumption, having a specific bedroom, maintaining sleep regularity, avoiding daytime naps and stress management, has been shown to be beneficial in people with psychosis [4,45], particularly if coupled with a portable sleep tracker [46]. This consists of a wearable tracker (Fitbit) that can detect sleep and physical activity. Such devices can be useful for increasing physical activity, self-awareness, motivation and goal setting.

Specific strategies targeting sleep deprivation in psychosis are increasingly available to not only improve sleep but also treat psychotic symptoms [38,47] and improve clinical outcomes and functional disability [48].

## 6. Biological and Other Pharmacological Treatments

Symptoms of insomnia include highly irregular patterns of sleep and wakefulness, difficulty falling asleep or staying asleep, nocturnal activity and excessive daytime impairment [49].

Several studies have shown that sleep problems are intrinsically linked to the cause and maintenance of psychotic symptoms [50,51] and to psychotic relapse [52]. People with psychosis and concurrent sleep–wake problems are also more likely to present cognitive impairments, depression, anxiety, reduced perceived coping skills, daytime dysfunction [53,54,55] and an increased risk of suicide [56]. Therefore, a high amount of research recommends that sleep disorders should be screened, monitored and treated according to DSM5 recommendations [3]. Studies have shown that interventions targeting insomnia symptoms can also lead to improvements in psychotic symptom severity, quality of life and functional outcomes.

Of the different pharmacological therapies for insomnia in ARSM or FEP, the most commonly used include the following:

(1) Standard pharmacology. Although empirical evidence regarding the sleep-promoting effects of antipsychotics and sedatives remains mixed, the vast majority of authors recommend the use of antipsychotic medications only to treat acute psychotic periods [57]. Waters and collaborators examined the relationship between the number of antipsychotic medications taken concurrently and the quality of sleep in a psychiatric inpatient population. Higher doses of antipsychotics were associated with better sleep, although they only had a relatively small effect on the improved sleep quality. These findings suggest that the use of antipsychotic medications with sedative properties has limited efficacy as a treatment option for sleep dysfunctions and that these medications are not a substitute for other interventions.
(2) Benzodiazepines and benzodiazepine receptor agonists (BzRAs). In 2019, Robertson et al. explored the current therapeutic options for the treatment of comorbid insomnia in schizophrenia [42]. Benzodiazepines and benzodiazepine receptor agonists (BzRAs) have been found to be the most commonly used drugs for the pharmacological treatment of insomnia [42]. Benzodiazepines reduce sleep latency and increase total sleep time with a rapid onset of action. They should be used with special caution in patients with psychosis as they have been correlated with a significant increase in mortality and because their long-term administration may result in attentional disturbances and reductions in working memory [42]. BzRAs have a safer therapeutic profile and more limited addictive properties compared to benzodiazepines [42].
(3) Orexin receptor antagonists. Suvorexant, an orexin receptor antagonist, has been found to reduce insomnia symptoms in a patient with schizophrenia after eight weeks of treatment. Other FDA-approved medication options indicated for the treatment of insomnia include barbiturates, ramelteon and doxepin [42].
(4) Melatonin (N-acetyl-5-methoxytryptamine) is an endogenous hormone secreted by the pineal gland that works by stabilizing circadian rhythms. It is used as a treatment for insomnia, jet lag and mood disorders and has shown few side effects. [48,58]. Melatonin treatment attenuated schizophrenia-like symptoms in mice and reduced histopathological alterations [59]. It has been correlated with an attenuation of behavioral deficits and a reduction in brain oxidative stress in a rodent model of schizophrenia. In humans, to the best of our knowledge, little evidence is available on the effects of melatonin on emerging psychosis.
(5) Other interventions (e.g., bright light therapy and independent sleep restriction therapy) exist, but there is insufficient evidence for their use in psychosis [60]. Table 2 and Table 3 summarize therapeutic strategies, both pharmacological and non-pharmacological, for ARMS and FEP individuals.

## 7. Conclusions

The possible mechanisms underlying the relationship between sleep and psychosis are complex, and their nature remains to be determined and better understood. Sleep disorders appear to contribute to cognitive/motor deficits in the ARMS period and may play a role in the etiology of psychosis.

Sleep disorders have been found to be associated with attenuated psychotic and other psychopathological symptoms in ARMS and to be implicated in the symptoms of FEP. As objective parameters, alterations in sleep architecture, which may be related to symptom severity and neurocognitive deficits, have been demonstrated. It has also been shown that sleep efficiency was lower in patients with FEP compared to a healthy population, with a lower spindle density in frontal areas, which can predict the severity of negative symptoms. In addition, the relationship between the measurements of REM sleep and BPRS suggest that REM sleep physiology may share common substrates with the symptoms of psychosis.

The most widely used non-pharmacological treatments for these sleep disorders include CBT for insomnia, acupuncture, physical exercise and basic sleep hygiene. CBT for insomnia is found to be the most effective therapy, although it is not the only one.

For biological treatments, benzodiazepines, antipsychotics and BzRAs are among the most commonly used pharmacological options. Other treatments, such as suvorexant, could be an option in the future, although further studies are needed as its benefits remain unclear at this stage.

Future studies are needed to fill the gap in our understanding regarding which treatment regimens work best for schizophrenia patients with comorbid insomnia. Larger scale clinical trials will also help to elucidate the extent to which effective insomnia treatment can lead to an improvement in schizophrenia symptoms and overall long-term clinical outcomes in this patient population.

These symptoms’ correlation with the transition to psychosis should be further investigated. Promoting sleep in this vulnerable population may be a promising intervention target to improve symptoms and functioning.

## Figures and Tables

**Table 1:** Characteristics of individuals with ARMSs.

APS	Attenuated Psychotic Symptoms	Individuals who have experienced subthreshold, attenuated forms of positive psychotic symptoms during the past year.
BLIPS	Brief Limited Intermittent Psychotic Symptoms	Individuals who have experienced episodes of frank psychotic symptoms that have not lasted longer than a week and have spontaneously abated.
Trait	Trait and State Risk Factor	Individuals with a first-degree relative with a psychotic disorder or who fulfill the criteria for schizotypal personality disorder and have presented a significant decrease in functioning during the previous year.

**Table 2:** Non-pharmacological strategies for the management of sleep disorders in ARMS and FEP patients.

Non-Pharmacological Treatment		
CBTi (Cognitive behavioral therapy for insomnia)	Reduction in insomnia and improvement in depression, anxiety and psychotic symptoms	[14,36,37,38,39]
Bright light therapy	Insufficient evidence	[60]
Cognitive restructuring techniques	Reduction in insomnia and nightmares at post-treatment, maintained at follow-up	[44]
Independent sleep restriction therapy	Insufficient evidence	[60]
Basic sleep hygiene education	Beneficial in people with psychosis	[4,45]
Provision of a portable sleep tracker	Beneficial in people with psychosis	[46]

**Table 3:** Other biological strategies for the management of sleep disorders in ARMS and FEP patients.

Biological Treatments		
Melatonin	Sedative and chronobiological effects with fewer side effects	[48,58]
Standard pharmacological (antipsychotic) and hypnotics	Only recommended for acute periods	[57]

## Data Availability

The data presented in this review are available upon request from the corresponding author.
